# Only true pelagics mix: comparative phylogeography of deepwater bathybatine cichlids from Lake Tanganyika

**DOI:** 10.1007/s10750-018-3752-3

**Published:** 2018-09-19

**Authors:** Stephan Koblmüller, Lukas Zangl, Christine Börger, Daniel Daill, Maarten P. M. Vanhove, Christian Sturmbauer, Kristina M. Sefc

**Affiliations:** 1 Institute of Biology, University of Graz, Universitätsplatz 2, 8010 Graz, Austria; 2 Institute of Vertebrate Biology, Academy of Sciences of the Czech Republic, Května 8, 603 65 Brno, Czech Republic; 3 Consultants in Aquatic Ecology and Engineering – blattfisch e.U., Gabelsbergerstraße 7, 4600 Wels, Austria; 4 Department of Botany and Zoology, Faculty of Science, Masaryk University, Kotlářská 2, 611 34 Brno, Czech Republic; 5 Research Group Zoology: Biodiversity & Toxicology, Centre for Environmental Sciences, Hasselt University, Agoralaan Gebouw D, 3590 Diepenbeek, Belgium; 6 Zoology Unit, Finnish Museum of Natural History, University of Helsinki, P.O. Box 17, 00014 Helsinki, Finland; 7 Laboratory of Biodiversity and Evolutionary Genomics, Department of Biology, University of Leuven, Ch. Deberiotstraat 32, 3000 Louvain, Belgium

**Keywords:** Cichlidae, *Bathybates*, *Hemibates*, Panmixis, Pelagic fishes, Phylogeography

## Abstract

In the absence of dispersal barriers, species with great dispersal ability are expected to show little, if at all, phylogeographic structure. The East African Great Lakes and their diverse fish faunas provide opportunities to test this hypothesis in pelagic fishes, which are presumed to be highly mobile and unrestricted in their movement by physical barriers. Here, we address the link between panmixis and pelagic habitat use by comparing the phylogeographic structure among four deepwater cichlid species of the tribe Bathybatini from Lake Tanganyika. We show that the mitochondrial genealogies (based on the most variable part or the control region) of the four species are very shallow (0.8–4% intraspecific divergence across entire distribution ranges) and that all species experienced recent population growth. A lack of phylogeographic structure in the two eupelagic species, *Bathybates fasciatus* and *B. leo*, was consistent with expectations and with findings in other pelagic cichlid species. Contrary to expectations, a clear phylogeographic structure was detected in the two benthopelagic species, *B. graueri* and *Hemibates stenosoma*. Differences in genetic diversity between eupelagic and benthopelagic species may be due to differences in their dispersal propensity, mediated by their respective predatory niches, rather than precipitated by external barriers to dispersal.

**Electronic supplementary material:**

The online version of this article (10.1007/s10750-018-3752-3) contains supplementary material, which is available to authorized users.

## Introduction

Contemporary patterns of genetic diversity and population connectivity within species are influenced by demographic history, historical and present barriers to gene flow, and the species’ active and/or passive dispersal ability (Hewitt, 2000; Ellegren & Galtier, 2016). Thus, highly vagile generalist species with great active dispersal ability typically show little phylogeographic structure, sometimes even across their entire distribution range (e.g., Koblmüller et al., 2012; Statham et al., 2014; Nebel et al., 2015; Pfeiler & Markow, 2017). This is particularly true for pelagic fishes, which are usually highly mobile with their dispersal not restricted by physical barriers (e.g., Graves & McDowell, 2003; Theisen et al., 2008; García-Rodríguez et al., 2011), even though exceptions have been reported (e.g., Perrin & Borsa, 2001; Lu et al., 2006; Fauvelot & Borsa, 2011; Sebastian et al., 2017).

Vast pelagic freshwater environments are found in the world’s largest lakes, including the East African Great Lakes, Tanganyika and Malawi, which are home to extraordinarily species-rich radiations of cichlid fishes (Fryer & Iles, 1972; Turner et al., 2001; Koblmüller et al., 2008; Salzburger et al., 2014). Even though most of the cichlid diversity is found in the littoral zone, a few lineages have successfully colonized and radiated in the pelagic and benthopelagic habitats (Turner et al., 2004; Koblmüller et al., 2008). Yet, what drives diversification in pelagic cichlids, what the ecological delineators among species are, and how these factors influence dispersal and gene flow, are still poorly understood. Niche partitioning according to food preferences or water depth has been suggested to have played a role (Coulter, 1991; Konings, 1998; Kirchberger et al., 2012; Hahn et al., 2017), as well as, at least for some Lake Malawi species, breeding-site fidelity (Genner et al., 2010a). It is generally assumed that, contrary to stenotopic littoral species which often show significant population differentiation even across minor habitat barriers (e.g., Rico & Turner, 2002; Sefc et al., 2017a), the eupelagic and benthopelagic species form panmictic populations across an entire lake. Previous studies indeed demonstrated this to be true for a few Lake Malawi species (*Diplotaxodon* spp.: Shaw et al., 2000, Genner et al., 2010a; *Rhamphochromis longiceps*: Günther, 1864, Genner et al., 2008) and one Lake Tanganyika species (*Boulengerochromis microlepis*: Boulenger, 1899, Koblmüller et al., 2015). It is unclear, however, whether this is indeed a general pattern.

Throughout the Pleistocene, faunal communities of Lakes Malawi and Tanganyika were heavily impacted by recurrent climatically induced lake level fluctuations (e.g., Cohen et al., 1997; McGlue et al., 2008; Lyons et al., 2015). Lake levels dropped (and rose) repeatedly by several hundreds of metres in these lakes, and these fluctuations are regarded as an important mechanism driving and synchronizing diversification within and across the lakes (Rossiter, 1995; Sturmbauer et al., 2001; Sefc et al., 2017b). Whereas Lake Malawi remained a single, although very shallow, lake during extreme lake level lowstands, the most dramatic lake level drops may have subdivided Lake Tanganyika into up to four paleolakes, corresponding with current subbasins (Danley et al., 2012; Sturmbauer et al., 2017). These events could potentially have facilitated allopatric diversification in pelagic and benthopelagic cichlids, which might be evident in patterns of speciation and current phylogeographic structure within species. Indeed, support for the influence of past separation(s) of the lake’s subbasins comes from the different compositions of their cichlid communities (Van Steenberge et al., 2011).

In the present study, we address the potential links between panmixis and pelagic habitat use by comparing the phylogeographic structure among four species of deepwater cichlids. In Lake Tanganyika, cichlids of the endemic tribe Bathybatini, together with *Lates* perches, large clariid catfishes, and the emperor cichlid (*Boulengerochromis microlepis*), are the dominant predators in the deep pelagic and benthopelagic habitats down to the limit of the oxygen--bearing layer (~ 50–200 m). Currently, this tribe includes seven *Bathybates* and two *Hemibates* species, one of which was described only recently (Schedel & Schliewen, 2017). All bathybatine cichlids feed predominantly on fish, are maternal mouthbrooders, and are sexually dichromatic with silvery females and males that exhibit conspicuous species-specific patterns of blackish spots and stripes on a silvery ground. Apart from the smallest species, *B. minor* Boulenger, 1906, which barely reaches a total length of 20 cm, all members of the tribe are large species exceeding 30 cm. The Bathybatini are an ancient lineage within Lake Tanganyika’s cichlid species flock (Salzburger et al., 2002; Koblmüller et al., 2005; Meyer et al., 2015; Takahashi & Sota, 2016; Irisarri et al., 2018), and it is assumed that their ancestors colonized the lake as one of the radiation’s seeding lineages (Salzburger et al., 2002). The phylogenetic relationships within the tribe are well established (Koblmüller et al., 2005; Kirchberger et al., 2012; Schedel & Schliewen, 2017). Yet, nothing is known about their phylogeographic or population genetic structure. Such data would increase our knowledge of factors and processes shaping intraspecific diversity in highly mobile (cichlid) fish species. In addition, they may also aid in identifying potentially segregated fish stocks, which is important for conservation and fisheries management. Indeed, some bathybatine species are heavily targeted by local fishermen. Currently, they do not appear to be under immediate threat of overfishing, but data on catch statistics do not exist for any of these species (Petit & Shipton, 2012).

Here, we characterize the genetic diversity of four bathybatine cichlid species—the eupelagic species *Bathybates fasciatus* Boulenger, 1901 and *B. leo* Poll, 1956 that live and prey in the open water zone, and the benthopelagic species *B. graueri* Steindachner, 1911 and *Hemibates stenosoma* (Boulenger, 1901) that live and prey above the bottom—and reconstruct their phylogeographic structure and demographic history based on mitochondrial DNA sequences. The findings are discussed in the light of the biological characteristics of the species and the hydrological history of Lake Tanganyika.

## Materials and methods

**Fig. 1:**
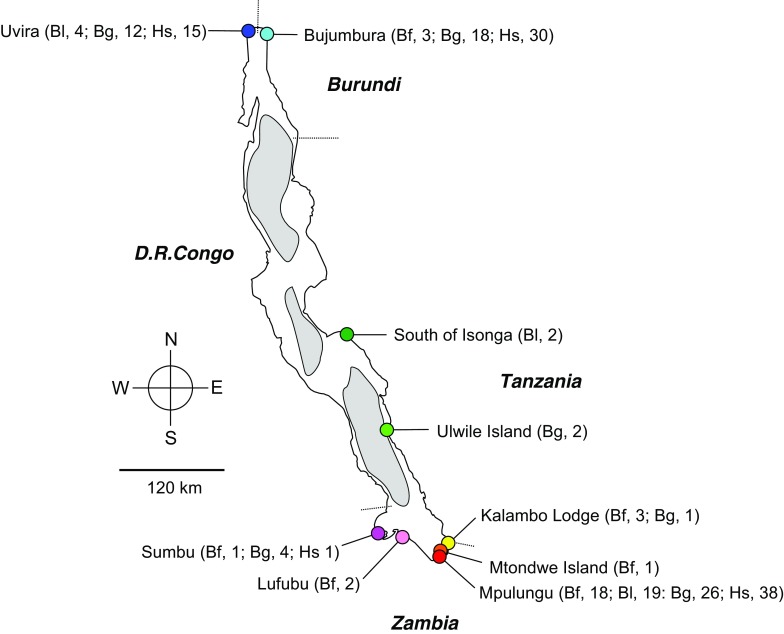
Map of Lake Tanganyika showing the sampling localities. Number of individual samples per species and locality are given in parentheses. Bf, *Bathybates fasciatus*; Bl, *Bathybates leo*; Bg, *Bathybates graueri*; Hs, *Hemibates stenosoma*

Fin clips were taken from 28, 63, 25, and 84 individuals of *Bathybates fasciatus, B. graueri, B. leo, and Hemibates stenosoma*, respectively, obtained at local fish markets in Bujumbura, Uvira and Mpulungu or from artisanal fishermen on the lake, during several field trips between 1992 and 2016 (Fig. 1, Supplementary Table 1), and preserved in 96% ethanol. Whole genomic DNA was extracted following a rapid Chelex protocol (Richlen & Barber, 2005). The most variable part of the mitochondrial control region was amplified and sequenced according to the protocols described in Koblmüller et al. (2011) and Duftner et al. (2005), respectively. The primers used for PCR and chain-termination sequencing were L-Pro-F_Tropheus (Koblmüller et al., 2011) and TDK-D (Lee et al., 1995). DNA fragments were purified with Sephadex™ G-50 (Amersham Biosciences) and visualized on an ABI 3130xl capillary sequencer (Applied Biosystems). Sequences were aligned by eye in MEGA7 (Kumar et al., 2016). The lengths of the final alignments were 354 bp for *B. fasciatus* and *B. graueri*, 355 bp for *B. leo*, and 320 bp for *H. stenosoma*. Sequences are deposited in GenBank under the accession numbers listed in Supplementary Table 1.

Haplotype (*H*_d_) and nucleotide diversity (*π*) were calculated in DnaSP 5.10 (Librado & Rozas, 2009). Intraspecific phylogenetic relationships among haplotypes were inferred by means of statistical parsimony networks (Templeton et al., 1992) in PopART (Leigh & Bryant, 2015). To test for signals of past population expansion, mismatch distributions were calculated in Arlequin 3.5.1.2 (Excoffier & Lischer, 2010). The fit of the observed mismatch distribution to the expectations based on growth parameter estimates was evaluated by the sum of squared differences (*SSD*) and the raggedness index (*rg*). In addition, past population size trajectories and time to the most recent common ancestor (tMRCA) were inferred by means of a Bayesian coalescent approach [Bayesian skyline plot (BSP) tree prior] as implemented in BEAST 1.8.4 (Drummond et al., 2012). We employed the model of molecular evolution selected by the Bayesian information criterion (BIC) in jModelTest 0.1 (Posada, 2008) and assumed a strict molecular clock and a substitution rate of 0.0325 and alternatively 0.057 per site per MY (Koblmüller et al., 2009). Two independent MCMC runs of one million generations each were conducted, sampling every 1000th step with a burn-in of the first 10% of sampled generations. Verification of effective sample sizes [ESS > 200 for all parameters, indicating that the parameter log file accurately reflected the posterior distribution (Kuhner, 2009)], trace of MCMC runs, and visualization of past demographic changes were done in Tracer 1.5 (Rambaut & Drummond, 2009).

## Results

**Fig. 2:**
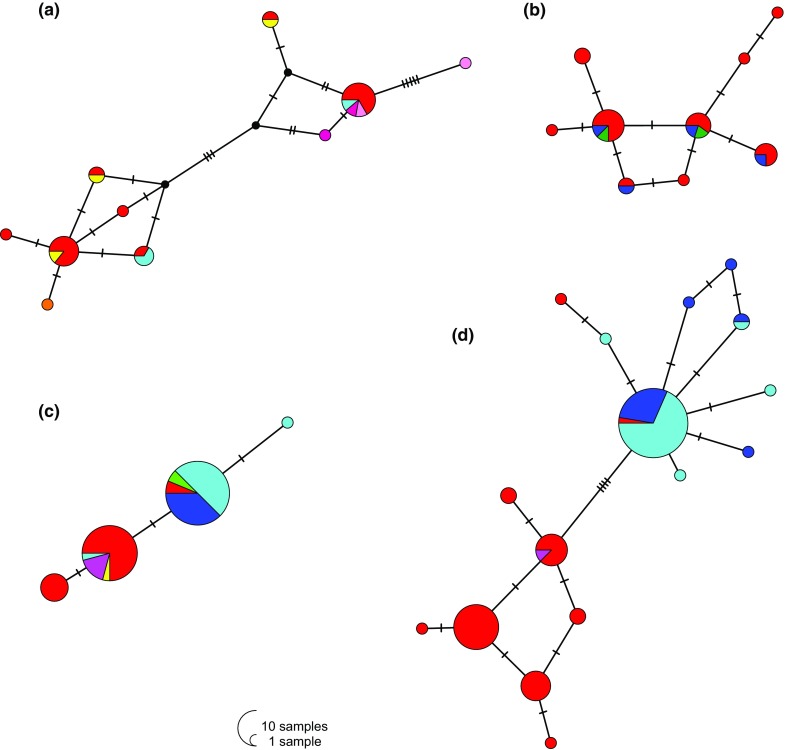
Statistical parsimony networks of **a** *Bathybates fasciatus*, **b** *Bathybates leo*, **c** *Bathybates graueri*, **d** *Hemibates stenosoma*. Circle sizes are proportional to haplotype frequency. Tick marks indicate the number of mutations between haplotypes. Different colors refer to different sampling localities as shown in Fig. 1

Genetic diversity was somewhat higher in the eupelagic species *B. fasciatus* and *B. leo* than in the benthopelagic species *B. graueri* and *H. stenosoma* (Table 1). The number of haplotypes found per species was 10 for *B. fasciatus*, 4 for *B. graueri*, 9 for *B. leo* and 16 for *H. stenosoma*. Intraspecific divergence varied among species, with maximum numbers of pairwise differences amounting to 14 in *B. fasciatus*, 4 in *B. leo*, 3 in *B. graueri* and 9 in *H. stenosoma*. Whereas the haplotype networks of the eupelagic species indicated no geographic structure (Fig. 2a, b), a clear separation into northern and southern haplotypes became evident in the benthopelagic *B. graueri* and *H. stenosoma* (Fig. 2c, d), despite some haplotype sharing between northern and southern samples. Specifically, in *B. graueri*, the dominant northern and southern haplotypes were also found in two southern and one northern individual(s), respectively (Fig. 2c), and in *H. stenosoma*, two southern individuals grouped within the northern clade (Fig. 2d).

**Table 1:** Sample sizes (*N*) and genetic diversity estimates for the four target species and distinct geographic clades of *Hemibates stenosoma*

Species	*N*	*H*	*H*_d_	*π*	Maximum intraspecific divergence (%)
*Bathybates fasciatus*	28	10	0.836	0.01330	4.0
*Bathybates graueri*	63	4	0.597	0.00202	0.8
*Bathybates leo*	25	9	0.847	0.00419	1.1
*Hemibates stenosoma* all	84	16	0.749	0.00933	2.5
*Hemibates stenosoma* North^a^	47	9	0.349	0.00143	1.1
*Hemibates stenosoma* South	37	7	0.743	0.00334	1.1

*H* number of haplotypes, *H*_d_ haplotype diversity, *π* nucleotide diversity

^a^This clade also includes two southern samples that cluster within this haplogroup

**Fig. 3:**
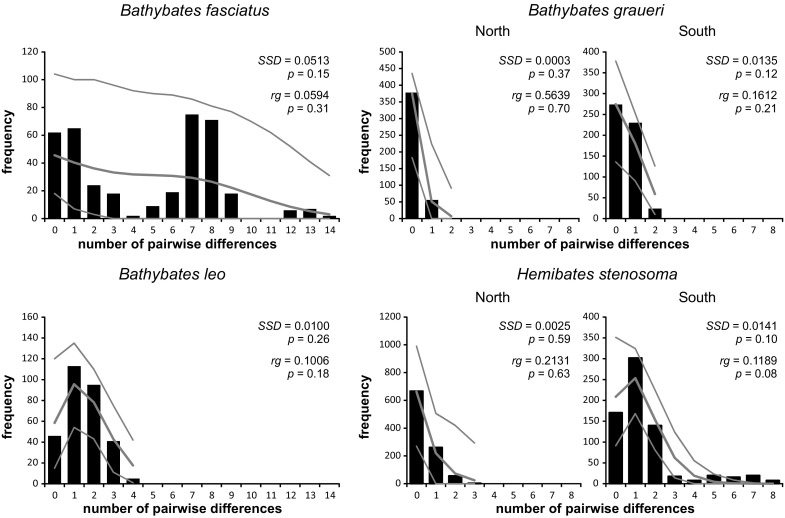
Mismatch distribution for the four target species and geographic clades within species (if applicable). Black columns represent the observed frequency of pairwise differences. Gray lines refer to the expected distribution based on parameter estimates and their 95% confidence limits simulated under a model of population growth. Sum of squared differences (*SSD*) and raggedness index (*rg*) and their respective *P* values are given to describe the fit of the observed mismatch distribution to the expectation based on growth parameter estimates

**Fig. 4:**
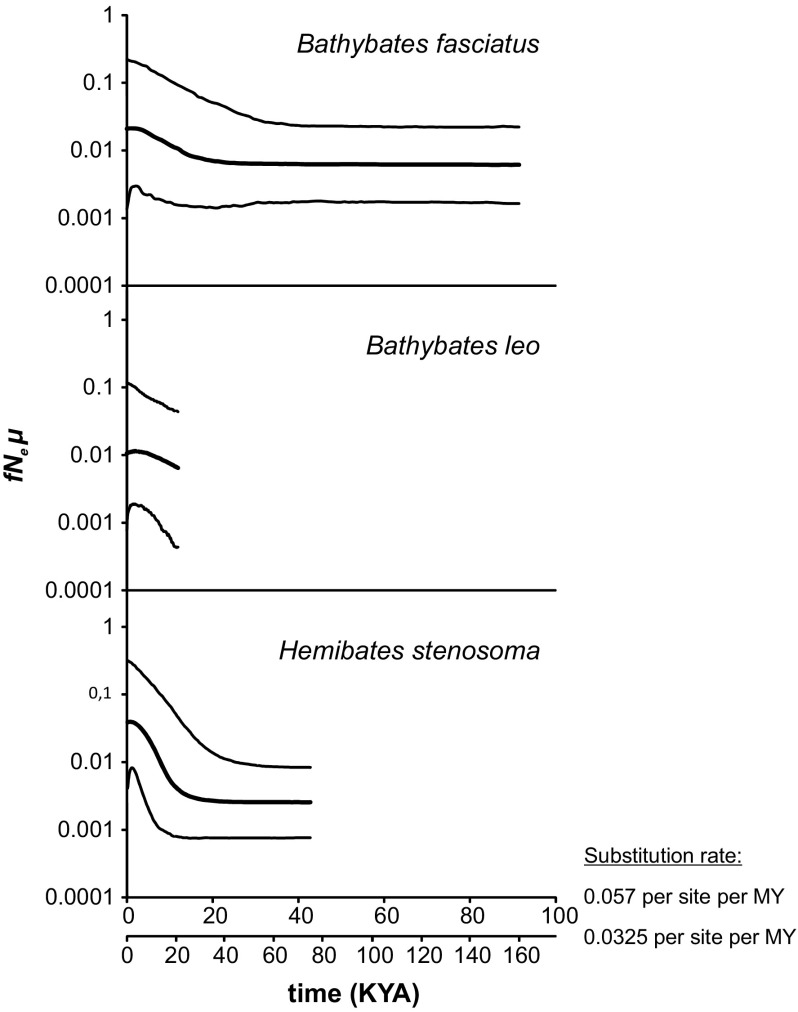
Bayesian skyline plots (BSP) of past population size trajectories assuming minimum and maximum substitution rates of 3.25 and 5.7% per site per MY (Koblmüller et al., 2009). Thick lines denote median estimate; thin lines indicate 95% highest posterior density (HPD) intervals. The y-axis represents the population size parameter (product of female effective population size, *fN*_e_, and mutation rate, *µ*)

Signatures of population expansion were detected in all four species. The fits of the observed mismatch distributions to the expectations based on growth parameter estimates, with nonsignificant *SSD* and *rg* values, indicated recent population growth in *B. fasciatus*, *B. leo*, both clades of *B. graueri* and the northern cade of *H. stenosoma* (Fig. 3). In the southern *H. stenosoma*, the presence of two divergent haplotypes caused a more ragged mismatch distribution. However, Bayesian skyline plot reconstructions produced clear evidence for strong recent growth in *H. stenosoma* (both clades pooled), as well as weaker expansions in *B. fasciatus* and *B. leo* (Fig. 3). The extremely low intraspecific divergence prevented the estimation of BSPs for *B. graueri*. The onset of the inferred recent population expansion was dated to about 20–40 KYA, depending on the substitution rate assumed, for *B. fasciatus* and *H. stenosoma*, but could not be estimated for *B. leo* because of low intraspecific divergence (Fig. 4). Estimates of the time to the most recent common ancestor differed among the four species and ranged from mean estimates of 22.6–39.7 KYA in *B. graueri* to 197.2–345.8 KYA in *B. fasciatus* (Table 2).

**Table 2:** Time to most recent common ancestor (tMRCA) of the four target species and the two distinct clades within *Hemibates stenosoma*, inferred based on minimum and maximum assumed substation rates of 3.25 and 5.7% per million years, respectively (Koblmüller et al., 2009)

Species	tMRCA (95% HPD)	
	3.25%	5.7%
*Bathybates fasciatus*	345,841 (160,409–555,496)	197,190 (91,461–316,730)
*Bathybates graueri*	39,705 (1,262–100,690)	22,639 (720–57,411)
*Bathybates leo*	84,884 (21,123–167,608)	48,399 (12,044–95,566)
*Hemibates stenosoma* all	220,704 (74,717–394,686)	125,840 (42,602–225,040)
*Hemibates stenosoma* North^a^	80,129 (22,191–155,887)	45,688 (12,653–88,883)
*Hemibates stenosoma* South	62,870 (19,054–123,116)	35,847 (10,864–70,768)

^a^This clade also includes two southern samples that cluster within this haplogroup

## Discussion

Analysis of the most variable region of the mitochondrial control region revealed a lack of phylogeographic structure in the two eupelagic species *B. fasciatus* and *B. leo*. This is consistent with findings in other pelagic species (e.g., Graves & McDowell, 2003; Theisen et al., 2008; García-Rodríguez et al., 2011) and the idea that the absence of physical barriers should preclude population structure in highly mobile species (Hartl & Clark, 1997). Haplotypes of the two benthopelagic species, *B. graueri* and *H. stenosoma*, in contrast, were divided into northern and southern clades. Sampling of all four species concentrated on the northernmost and southernmost regions of Lake Tanganyika (Fig. 1) and therefore spanned the largest possible distance (> 700 km) across the lake. Lacking samples from intermittent locations, it is not possible to assess whether the clades of northern and southern *B. graueri* and *H. stenosoma* represent the endpoints of phylogeographic isolation-by-distance continua or reflect the existence of discrete phylogeographic units. Haplotype sharing between northern and southern clades could therefore indicate either step-wise short-range or occasional long-range gene flow. Alternatively, given the shallow genetic divergence among clades, particularly in *B. graueri*, haplotype sharing may simply be a remnant of incomplete lineage sorting.

The observed phylogeographic structure in *B. graueri* and *H. stenosoma* contradicts the assumption that all *Bathybates* and *Hemibates* species form panmictic populations across the entire lake (Konings, 1998), and is surprising given the absence of apparent physical barriers to dispersal for benthopelagic species. Therefore, one must implicate ecological distinctions as dispersal restrictions. One potential explanation for the difference in large-scale phylogeographic patterns between the eu- and the benthopelagic bathybatine species might lie in their specialization on different types of prey. In pursuit of pelagic prey—mainly the lake’s two endemic clupeid species (Coulter, 1991)—*Bathybates fasciatus* and *B. leo*—roam the lakes’ pelagic zone down to the limit of the oxygen--bearing layer (~ 50 m in the north and ~ 200 m in the south of the lake). This specialization on pelagic prey requires these two species to be highly mobile and to move long distances through open water. *Bathybates graueri* and *H. stenosoma* are also mainly found at great depth, but they prey upon benthic and benthopelagic cichlids, in particular the various deepwater *Xenotilapia, Limnochromis* and *Trematocara* species (Coulter, 1991). Although some of these prey species, especially *Trematocara* spp., migrate to shallow waters during night, with *H. stenosoma* and probably also *B. graueri* in their wake (Coulter, 1991; Konings, 1998), these two predators do not need to move long distances through open water to find their prey. These differences in foraging behavior might translate into different dispersal patterns and result in range-wide admixture of the eupelagic hunters versus restricted gene flow in the benthopelagic ones. This contrast cannot be generalized, however: a study of another benthopelagic species from Lake Tanganyika, *Boulengerochromis microlepis*, showed that a benthopelagic life style per se does not necessarily imply phylogeographic structuring (Koblmüller et al., 2015). Thus, there might be additional ecological features that contribute to dispersal.

Maximum intraspecific divergence varied among the four Bathybatini species from 0.8 to 4% (Table 1), but was comparable to the lake-wide divergence observed in the benthopelagic *Boulengerochromis microlepis* (2.5%, Koblmüller et al., 2015). These divergence estimates are low in comparison to stenotopic littoral species of Lake Tanganyika (Duftner et al., 2007; Koblmüller et al., 2017). Similar values (2.2–4.2%) were estimated within individual populations of stenotopic littoral species (Duftner et al., 2006; Koblmüller et al., 2007, 2009; Sefc et al., 2007). So far, among Lake Tanganyika cichlids, the pattern of large intraspecific divergence in geographically highly structured littoral species versus low intraspecific divergence in highly vagile eu- and benthopelagic deepwater species has been confirmed without exception.

Recent population expansion has turned out as a commonality among the cichlids of Lake Malawi and Tanganyika (littoral, e.g., Genner et al., 2010b; Koblmüller et al., 2011; Genner & Turner, 2015; Husemann et al., 2015; Sturmbauer et al., 2017; Winkelmann et al., 2017; deepwater: Genner & Turner, 2015; Koblmüller et al., 2015). In littoral species, demographic fluctuations are expected to be tied-up with habitat shrinkage and expansion during lake level fluctuations, and indeed, the reconstructed population expansions are temporally aligned with the most recent lake level rises after the last glacial maximum (McGlue et al., 2008). The demographic histories of eu- and benthopelagic species have been assumed to be less impacted by lake level fluctuations. In fact, population growth in these species was typically not as strong as in littoral cichlids and occurred earlier (Genner & Turner, 2015; Koblmüller et al., 2015), which suggests that only the most severe lake level fluctuation had a significant impact on the population size trajectories of eu- and benthopelagic species. In contrast, the dating of the population expansions of the bathybatine cichlid species studied here is more similar to that in littoral Lake Tanganyika cichlids (e.g., Koblmüller et al., 2011, 2017; Sefc et al., 2017a; Sturmbauer et al., 2017; Winkelmann et al., 2017). As in the previous studies, our time estimates are subject to the time dependency of the molecular clock (Ho et al., 2007) and uncertainty about substitution rates and appropriate calibration points (discussed in Koblmüller et al., 2017). However, since estimates from the various cichlid species were obtained under similar conditions, these values can be readily compared across species. Intriguingly, signatures of recent population expansion were also detected in a monogenean gill parasite of *Bathybates* and *Hemibates* (Kmentová et al., 2016), suggesting concurrent population expansion in hosts and parasites.

In summary, we show that the mitochondrial genealogies of *B. fasciatus, B. graueri, B. leo and H. stenosoma* are very shallow and that all species experienced recent population growth. A clear phylogeographic structure is present only in the benthopelagic species *B. graueri* and *H. stenosoma*. Differences in genetic diversity between eu- and benthopelagic species may be due to differences in their dispersal propensity, mediated by their respective predatory niches, rather than different physical barriers to dispersal.

## Electronic supplementary material

Below is the link to the electronic supplementary material.

Supplementary material 1 (PDF 185 kb)
