# Supplementary material for: Only true pelagics mix: comparative phylogeography of deepwater bathybatine cichlids from Lake Tanganyika
Source: Hydrobiologia. 2018 Sep 19;832(1):93–103. doi: 10.1007/s10750-018-3752-3 (PMC6394743; doi:10.1007/s10750-018-3752-3)
Supplement: Supplementary file 1 — Supplementary material 1 (PDF 185 kb) [file 10750_2018_3752_MOESM1_ESM.pdf]

Supplementary Table 1 Sample IDs, sampling localities plus coordinates, GenBank accession numbers and sampling years.

SampleID	Locality	Coordinates	GenBank Acc. No.	Sampling year
Bathybates fasciatus				
1757	Lufubu	8°32’ S, 20°44’ E	AY663778*	1999
1772	Lufubu	8°32’ S, 20°44’ E	AY663779*	1999
2232	Mpulungu	8°46’ S, 31°06’ E	AY663780*	2001
12879	Mpulungu	8°46’ S, 31°06’ E	MH891864	2010
12884	Mpulungu	8°46’ S, 31°06’ E	MH891865	2010
12885	Mpulungu	8°46’ S, 31°06’ E	MH891866	2010
12889	Kalambo Lodge	8°37’ S, 31°12’ E	MH891867	2010
12890	Kalambo Lodge	8°37’ S, 31°12’ E	MH891868	2010
12891	Sumbu	8°31’ S, 30°29’ E	MH891869	2010
14727	Kalambo Lodge	8°37’ S, 31°12’ E	MH891870	2010
14730	Mpulungu	8°46’ S, 31°06’ E	MH891871	2010
14731	Mpulungu	8°46’ S, 31°06’ E	MH891872	2010
14930	Mtondwe Island	8°42‘ S, 31°07‘ E	MH891873	2012
14932	Bujumbura	3°23‘ S, 29°21‘ E	MH891875	2013
14933	Bujumbura	3°23‘ S, 29°21‘ E	MH891874	2013
15385	Bujumbura	3°23‘ S, 29°21‘ E	MH891876	2013
T16/1A1	Mpulungu	8°46’ S, 31°06’ E	MH891877	2016
T16/1A2	Mpulungu	8°46’ S, 31°06’ E	MH891878	2016
T16/1A4	Mpulungu	8°46’ S, 31°06’ E	MH891879	2016
T16/1A5	Mpulungu	8°46’ S, 31°06’ E	MH891880	2016
T16/1A6	Mpulungu	8°46’ S, 31°06’ E	MH891881	2016
T16/1A8	Mpulungu	8°46’ S, 31°06’ E	MH891882	2016
T16/1B4	Mpulungu	8°46’ S, 31°06’ E	MH891883	2016
T16/1B5	Mpulungu	8°46’ S, 31°06’ E	MH891884	2016
T16/1B10	Mpulungu	8°46’ S, 31°06’ E	MH891885	2016
T16/1C1	Mpulungu	8°46’ S, 31°06’ E	MH891886	2016
T16/1C2	Mpulungu	8°46’ S, 31°06’ E	MH891887	2016
T16/1C4	Mpulungu	8°46’ S, 31°06’ E	MH891888	2016
Bathybates leo				
1758	Mpulungu	8°46’ S, 31°06’ E	AY663774*	2001
2225	Mpulungu	8°46’ S, 31°06’ E	AY663775*	2003
2226	Mpulungu	8°46’ S, 31°06’ E	AY663776*	2003
2230	Mpulungu	8°46’ S, 31°06’ E	AY663777*	2003
12921	Mpulungu	8°46’ S, 31°06’ E	MH891889	2003
12922	Mpulungu	8°46’ S, 31°06’ E	MH891890	2003
12923	Mpulungu	8°46’ S, 31°06’ E	MH891891	2003
14924	Uvira	3°21‘ S, 29°11‘ E	MH891892	2013
14925	Uvira	3°21‘ S, 29°11‘ E	MH891893	2013
14926	Uvira	3°21‘ S, 29°11‘ E	MH891894	2013
14935	Uvira	3°21‘ S, 29°11‘ E	MH891895	2013
14937	South of Isonga	6°29‘ S, 30°10‘ E	MH891896	2007
14939	South of Isonga	6°29‘ S, 30°10‘ E	MH891897	2007
T16/1A3	Mpulungu	8°46’ S, 31°06’ E	MH891898	2016
T16/1A7	Mpulungu	8°46’ S, 31°06’ E	MH891899	2016
T16/1A9	Mpulungu	8°46’ S, 31°06’ E	MH891900	2016
T16/1A10	Mpulungu	8°46’ S, 31°06’ E	MH891901	2016
T16/1B1	Mpulungu	8°46’ S, 31°06’ E	MH891902	2016
T16/1B2	Mpulungu	8°46’ S, 31°06’ E	MH891903	2016

T16/1B3	Mpulungu	8°46’ S, 31°06’ E	MH891904	2016
T16/1B6	Mpulungu	8°46’ S, 31°06’ E	MH891905	2016
T16/1B7	Mpulungu	8°46’ S, 31°06’ E	MH891906	2016
T16/1B8	Mpulungu	8°46’ S, 31°06’ E	MH891907	2016
T16/1C3	Mpulungu	8°46’ S, 31°06’ E	MH891908	2016
T16/1C6	Mpulungu	8°46’ S, 31°06’ E	MH891909	2016
Bathybates graueri				
1760	Sumbu	8°31’ S, 30°29’ E	AY663768*	2001
1885	Ulwile	7°27‘ S, 30°34‘ E	AY663769*	1992
1886	Ulwile	7°27‘ S, 30°34‘ E	AY663770*	1992
2228	Mpulungu	8°46’ S, 31°06’ E	AY663771*	2003
12877	Mpulungu	8°46’ S, 31°06’ E	MH891910	2010
12878	Mpulungu	8°46’ S, 31°06’ E	MH891911	2010
12883	Mpulungu	8°46’ S, 31°06’ E	MH891912	2010
12892	Mpulungu	8°46’ S, 31°06’ E	MH891913	2010
12893	Mpulungu	8°46’ S, 31°06’ E	MH891914	2010
12894	Mpulungu	8°46’ S, 31°06’ E	MH891915	2010
12895	Mpulungu	8°46’ S, 31°06’ E	MH891916	2010
12896	Mpulungu	8°46’ S, 31°06’ E	MH891917	2010
12898	Mpulungu	8°46’ S, 31°06’ E	MH891918	2010
12899	Mpulungu	8°46’ S, 31°06’ E	MH891919	2010
12900	Mpulungu	8°46’ S, 31°06’ E	MH891920	2010
12901	Mpulungu	8°46’ S, 31°06’ E	MH891921	2010
12902	Mpulungu	8°46’ S, 31°06’ E	MH891922	2010
12919	Sumbu	8°31’ S, 30°29’ E	MH891923	2001
14708	Bujumbura	3°23‘ S, 29°21‘ E	MH891924	2013
14709	Bujumbura	3°23‘ S, 29°21‘ E	MH891925	2013
14710	Bujumbura	3°23‘ S, 29°21‘ E	MH891926	2013
14711	Bujumbura	3°23‘ S, 29°21‘ E	MH891927	2013
14712	Bujumbura	3°23‘ S, 29°21‘ E	MH891928	2013
14713	Bujumbura	3°23‘ S, 29°21‘ E	MH891929	2013
14714	Bujumbura	3°23‘ S, 29°21‘ E	MH891930	2013
14715	Bujumbura	3°23‘ S, 29°21‘ E	MH891931	2013
14717	Sumbu	8°31’ S, 30°29’ E	MH891932	2001
14728	Kalambo Lodge	8°37’ S, 31°12’ E	MH891933	2010
14729	Sumbu	8°31’ S, 30°29’ E	MH891934	2010
14732	Mpulungu	8°46’ S, 31°06’ E	MH891935	2010
14734	Mpulungu	8°46’ S, 31°06’ E	MH891936	2010
14735	Mpulungu	8°46’ S, 31°06’ E	MH891937	2010
14736	Mpulungu	8°46’ S, 31°06’ E	MH891938	2010
14737	Mpulungu	8°46’ S, 31°06’ E	MH891939	2010
14739	Mpulungu	8°46’ S, 31°06’ E	MH891940	2010
14740	Mpulungu	8°46’ S, 31°06’ E	MH891941	2010
14741	Bujumbura	3°23‘ S, 29°21‘ E	MH891942	2013
14742	Bujumbura	3°23‘ S, 29°21‘ E	MH891943	2013
14748	Mpulungu	8°46’ S, 31°06’ E	MH891944	2010
14749	Mpulungu	8°46’ S, 31°06’ E	MH891945	2010
14750	Mpulungu	8°46’ S, 31°06’ E	MH891946	2010
14758	Uvira	3°21‘ S, 29°11‘ E	MH891947	2013
14760	Uvira	3°21‘ S, 29°11‘ E	MH891948	2013
14764	Uvira	3°21‘ S, 29°11‘ E	MH891949	2013
14765	Uvira	3°21‘ S, 29°11‘ E	MH891950	2013
14766	Uvira	3°21‘ S, 29°11‘ E	MH891951	2013

14767	Uvira	3°21‘ S, 29°11‘ E	MH891952	2013
14768	Uvira	3°21‘ S, 29°11‘ E	MH891953	2013
14769	Uvira	3°21‘ S, 29°11‘ E	MH891954	2013
14770	Uvira	3°21‘ S, 29°11‘ E	MH891955	2013
14771	Uvira	3°21‘ S, 29°11‘ E	MH891956	2013
14772	Uvira	3°21‘ S, 29°11‘ E	MH891957	2013
14774	Bujumbura	3°23‘ S, 29°21‘ E	MH891958	2013
14775	Bujumbura	3°23‘ S, 29°21‘ E	MH891959	2013
14776	Bujumbura	3°23‘ S, 29°21‘ E	MH891960	2013
14777	Bujumbura	3°23‘ S, 29°21‘ E	MH891961	2013
14778	Bujumbura	3°23‘ S, 29°21‘ E	MH891962	2013
14779	Bujumbura	3°23‘ S, 29°21‘ E	MH891963	2013
14936	Uvira	3°21‘ S, 29°11‘ E	MH891964	2013
15382	Bujumbura	3°23‘ S, 29°21‘ E	MH891965	2013
15383	Bujumbura	3°23‘ S, 29°21‘ E	MH891966	2013
T16/1C5	Mpulungu	8°46’ S, 31°06’ E	MH891967	2016
T16/1C7	Mpulungu	8°46’ S, 31°06’ E	MH891968	2016
Hemibates stenosoma				
1759	Sumbu	8°31’ S, 30°29’ E	AY663761[*]	1999
1760	Mpulungu	8°46’ S, 31°06’ E	AY663762[*]	2001
2234	Mpulungu	8°46’ S, 31°06’ E	AY663764[*]	2003
14523	Mpulungu	8°46’ S, 31°06’ E	MH891969	2006
14525	Mpulungu	8°46’ S, 31°06’ E	MH891970	2006
14527	Mpulungu	8°46’ S, 31°06’ E	MH891971	2006
14528	Mpulungu	8°46’ S, 31°06’ E	MH891972	2006
14529	Mpulungu	8°46’ S, 31°06’ E	MH891973	2006
14530	Mpulungu	8°46’ S, 31°06’ E	MH891974	2006
14531	Mpulungu	8°46’ S, 31°06’ E	MH891975	2006
14532	Mpulungu	8°46’ S, 31°06’ E	MH891976	2006
14534	Mpulungu	8°46’ S, 31°06’ E	MH891977	2006
14538	Mpulungu	8°46’ S, 31°06’ E	MH891978	2006
14539	Mpulungu	8°46’ S, 31°06’ E	MH891979	2006
14540	Mpulungu	8°46’ S, 31°06’ E	MH891980	2006
14542	Mpulungu	8°46’ S, 31°06’ E	MH891981	2006
14544	Mpulungu	8°46’ S, 31°06’ E	MH891982	2006
14549	Mpulungu	8°46’ S, 31°06’ E	MH891983	2007
14551	Mpulungu	8°46’ S, 31°06’ E	MH891984	2007
14553	Bujumbura	3°23‘ S, 29°21‘ E	MH891985	2013
14554	Bujumbura	3°23‘ S, 29°21‘ E	MH891986	2013
14555	Bujumbura	3°23‘ S, 29°21‘ E	MH891987	2013
14556	Bujumbura	3°23‘ S, 29°21‘ E	MH891988	2013
14557	Bujumbura	3°23‘ S, 29°21‘ E	MH891989	2013
14558	Bujumbura	3°23‘ S, 29°21‘ E	MH891990	2013
14559	Bujumbura	3°23‘ S, 29°21‘ E	MH891991	2013
14560	Bujumbura	3°23‘ S, 29°21‘ E	MH891992	2013
14561	Bujumbura	3°23‘ S, 29°21‘ E	MH891993	2013
14562	Bujumbura	3°23‘ S, 29°21‘ E	MH891994	2013
14563	Bujumbura	3°23‘ S, 29°21‘ E	MH891995	2013
14564	Bujumbura	3°23‘ S, 29°21‘ E	MH891996	2013
14565	Bujumbura	3°23‘ S, 29°21‘ E	MH891997	2013
14566	Bujumbura	3°23‘ S, 29°21‘ E	MH891998	2013
14567	Bujumbura	3°23‘ S, 29°21‘ E	MH891999	2013
14568	Bujumbura	3°23‘ S, 29°21‘ E	MH892000	2013

14569	Bujumbura	3°23‘ S, 29°21‘ E	MH892001	2013
14570	Bujumbura	3°23‘ S, 29°21‘ E	MH892002	2013
14571	Bujumbura	3°23‘ S, 29°21‘ E	MH892003	2013
14572	Bujumbura	3°23‘ S, 29°21‘ E	MH892004	2013
14573	Bujumbura	3°23‘ S, 29°21‘ E	MH892005	2013
14574	Bujumbura	3°23‘ S, 29°21‘ E	MH892006	2013
14575	Bujumbura	3°23‘ S, 29°21‘ E	MH892007	2013
14576	Bujumbura	3°23‘ S, 29°21‘ E	MH892008	2013
14577	Bujumbura	3°23‘ S, 29°21‘ E	MH892009	2013
14578	Bujumbura	3°23‘ S, 29°21‘ E	MH892010	2013
14579	Bujumbura	3°23‘ S, 29°21‘ E	MH892011	2013
14580	Bujumbura	3°23‘ S, 29°21‘ E	MH892012	2013
14581	Bujumbura	3°23‘ S, 29°21‘ E	MH892013	2013
14582	Uvira	3°21‘ S, 29°11‘ E	MH892014	2013
14583	Uvira	3°21‘ S, 29°11‘ E	MH892015	2013
14584	Uvira	3°21‘ S, 29°11‘ E	MH892016	2013
14585	Uvira	3°21‘ S, 29°11‘ E	MH892017	2013
14586	Uvira	3°21‘ S, 29°11‘ E	MH892018	2013
14587	Uvira	3°21‘ S, 29°11‘ E	MH892019	2013
14588	Uvira	3°21‘ S, 29°11‘ E	MH892020	2013
14589	Uvira	3°21‘ S, 29°11‘ E	MH892021	2013
14590	Uvira	3°21‘ S, 29°11‘ E	MH892022	2013
14591	Uvira	3°21‘ S, 29°11‘ E	MH892023	2013
14592	Uvira	3°21‘ S, 29°11‘ E	MH892024	2013
14593	Uvira	3°21‘ S, 29°11‘ E	MH892025	2013
14594	Uvira	3°21‘ S, 29°11‘ E	MH892026	2013
14595	Uvira	3°21‘ S, 29°11‘ E	MH892027	2013
14596	Uvira	3°21‘ S, 29°11‘ E	MH892028	2013
14597	Uvira	3°21‘ S, 29°11‘ E	MH892029	2013
T16/1C9	Mpulungu	8°46’ S, 31°06’ E	MH892030	2016
T16/1C10	Mpulungu	8°46’ S, 31°06’ E	MH892031	2016
T16/1D1	Mpulungu	8°46’ S, 31°06’ E	MH892032	2016
T16/1D2	Mpulungu	8°46’ S, 31°06’ E	MH892033	2016
T16/1D3	Mpulungu	8°46’ S, 31°06’ E	MH892034	2016
T16/1D4	Mpulungu	8°46’ S, 31°06’ E	MH892035	2016
T16/1D5	Mpulungu	8°46’ S, 31°06’ E	MH892036	2016
T16/1D6	Mpulungu	8°46’ S, 31°06’ E	MH892037	2016
T16/1D7	Mpulungu	8°46’ S, 31°06’ E	MH892038	2016
T16/1D8	Mpulungu	8°46’ S, 31°06’ E	MH892039	2016
T16/1D9	Mpulungu	8°46’ S, 31°06’ E	MH892040	2016
T16/1D10	Mpulungu	8°46’ S, 31°06’ E	MH892041	2016
T16/1E1	Mpulungu	8°46’ S, 31°06’ E	MH892042	2016
T16/1E2	Mpulungu	8°46’ S, 31°06’ E	MH892043	2016
T16/1E3	Mpulungu	8°46’ S, 31°06’ E	MH892044	2016
T16/1E4	Mpulungu	8°46’ S, 31°06’ E	MH892045	2016
T16/1E5	Mpulungu	8°46’ S, 31°06’ E	MH892046	2016
T16/1E6	Mpulungu	8°46’ S, 31°06’ E	MH892047	2016
T16/1E8	Mpulungu	8°46’ S, 31°06’ E	MH892048	2016
T16/1E9	Mpulungu	8°46’ S, 31°06’ E	MH892049	2016

Note, that samples from Mpulungu, Bujumbura and Uvira were obtained at local fish markets. Hence, these samples originate from anywhere near these towns and the respective coordinates should be considered as rough approximations.

*, previously published sequences (Koblmüller et al., 2005).
